# Age-related dysfunction of the DNA damage response in intestinal stem cells

**DOI:** 10.1186/s41232-019-0096-y

**Published:** 2019-04-26

**Authors:** Koichiro Watanabe, Yasuaki Ikuno, Yumi Kakeya, Shinsuke Ikeno, Hitomi Taniura, Masayoshi Kurono, Keito Minemori, Yu Katsuyama, Hayato Naka-Kaneda

**Affiliations:** Department of Anatomy, Shiga University of Medical Science, Seta Tsukinowa-cho, Otsu, Shiga 520-2192 Japan

**Keywords:** Aging, Intestinal stem cell, DNA damage response

## Abstract

**Background:**

Senescence increases the risks of inflammatory bowel diseases and colon cancer. Intestinal stem cells (ISCs) in crypts differentiate into epithelial cells and thereby maintain intestinal homeostasis. However, the influence of aging on the functions of ISCs is largely unknown. The mutation rate is highest in the small and large intestines. Numerous types of naturally occurring DNA damage are removed by the DNA damage response (DDR). This response induces DNA repair and apoptosis; therefore, its dysregulation leads to accumulation of damaged DNA and consequently cellular dysfunctions, including tumorigenesis. This study investigated whether aging affects the DDR in mouse ISCs.

**Methods:**

Young (2–3-month-old) and old (> 19-month-old) Lgr5-EGFP-IRES-creERT2 mice were irradiated. The DDR in Lgr5-positive ISCs was compared between these mice by immunohistochemical analyses.

**Results:**

Induction of DDR marker proteins (phosphorylated ATR and 53BP1), inflammatory factors (phosphorylated NF-κB and interleukin-6), and a mitochondrial biogenesis-associated gene (peroxisome proliferator-activated receptor-γ coactivator 1α) was lower in old ISCs than in young ISCs in vivo.

**Conclusion:**

The competence of the DDR in ISCs declines with age in vivo.

**Electronic supplementary material:**

The online version of this article (10.1186/s41232-019-0096-y) contains supplementary material, which is available to authorized users.

## Background

Inflammatory bowel diseases (IBDs), such as ulcerative colitis and Crohn’s disease, are conventionally regarded as early-onset diseases. However, elderly-onset IBDs were recently reported, and the elderly population is growing worldwide [1]. Aging is related to disruption of tissue homeostasis, which increases the risks of developing IBDs and colon cancer. However, the molecular mechanisms underlying this process are largely unknown. Various age-related dysfunctions of adult tissue-resident stem/progenitor cells (TSCs, also known as somatic stem cells) are associated with perturbation of tissue homeostasis [2]. Restoration of stem cell functions has attracted much attention as a promising therapeutic strategy for geriatric diseases [3–7]. The intestinal epithelium is one of the most rapidly renewing tissues in the body. Lgr5-expressing intestinal stem cells (ISCs) in crypts differentiate into epithelial cells and thereby maintain intestinal homeostasis [8]. Therefore, dysfunction of ISCs may be important for the disruption of intestinal homeostasis and subsequent induction of functional disorders. However, the influence of aging on the functions of ISCs and induction of diseases is largely unknown.

Recent studies demonstrated that accumulation of senescent cells promotes organismal aging and that senolytics improve age-associated phenotypes and prolong lifespans [9–13]. Senescent cells secrete a myriad of inflammatory factors, a phenomenon termed the senescence-associated secretory phenotype (SASP), to recruit immune cells for eliminating themselves and thereby prevent their malignant transformation via the immune senescence surveillance system [14, 15]. Cells become senescent in response to various aging stresses, such as oxidative stress, telomere shortening, inflammation, irradiation, exposure to chemicals, and the mitotic stress, all of which induce DNA damage [16]. Numerous types of DNA damage occur naturally and are removed by the DNA damage response (DDR). This response induces DNA repair and apoptosis; therefore, its dysregulation leads to accumulation of damaged DNA and consequently cellular dysfunctions, including tumorigenesis [17, 18]. The mutation rate is highest in the small and large intestines [19]. However, the influence of aging on the DDR in ISCs has not been studied. Here, we compared induction of the DDR, inflammation, and mitochondrial biogenesis upon irradiation between young and old mouse ISCs in vivo.

## Methods

### Induction of the DDR

Young (2–3-month-old) and old (> 19-month-old) Lgr5-EGFP-IRES-creERT2 mice (The Jackson Laboratory) were total-body irradiated with or without 10 Gy of X-rays and sacrificed 8 h later. Swiss roll sections of paraffin-embedded intestines were prepared and immunohistochemically analyzed. The list of mice is shown in Additional file 1: Table S1.

### Immunohistochemistry

Intestines were fixed in phosphate-buffered saline containing 4% formaldehyde overnight and rolled. Swiss roll sections of paraffin-embedded intestines were prepared, subjected to heat-induced antigen retrieval with citrate buffer (pH 6.0) at 121 °C for 20 min, and then stained with antibodies against phosphorylated ataxia telangiectasia and Rad3 related (pATR; 1:200, GeneTex, GTX128145), transformation related protein 53 binding protein 1 (53BP1; 1:1000, Abcam, ab36823), phosphorylated v-rel reticuloendotheliosis viral oncogene homolog A (pNF-κB; 1:200, Abcam, ab86299), interleukin (IL)-6 (1:400, Abcam, ab6672), peroxisome proliferator-activated receptor-γ coactivator 1α (PGC-1α; 1:250, Abcam, ab54481), and enhanced green fluorescent protein (GFP; 1:1000, Aves Labs, GFP-1020). Jejunal regions of crypts were analyzed using a microscope (Olympus, IX83).

### Statistical analyses

Three independent experiments were included in each statistical analysis. Statistical significance was determined by two-tailed *t* tests. Throughout the study, *p* values < 0.05 were considered statistically significant.

## Results

### The DDR is perturbed in old ISCs

**Fig. 1:**
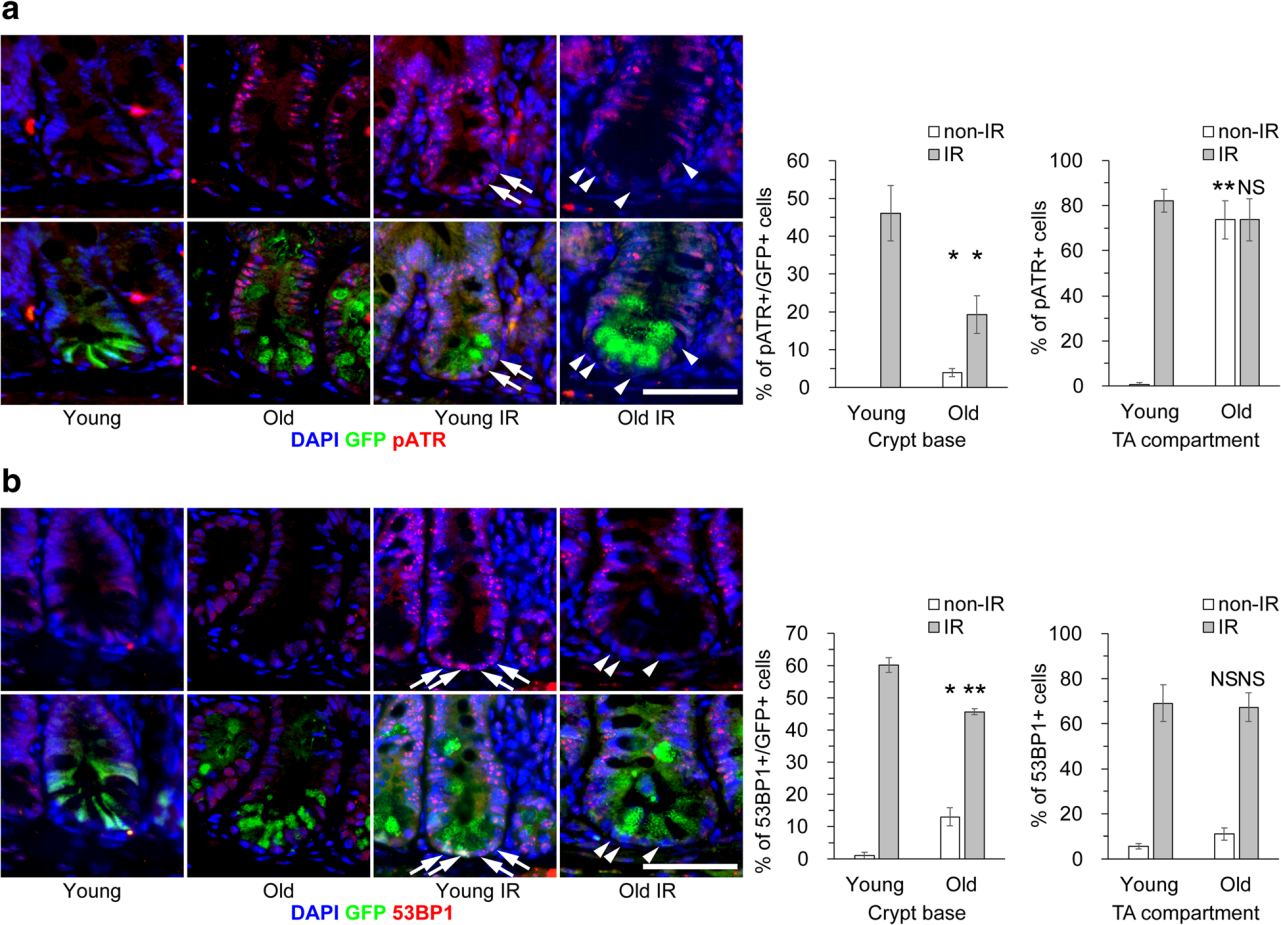
The DDR is perturbed in old ISCs. Immunohistochemical analyses of DDR marker proteins were performed in young and old jejunum with (IR) or without 10 Gy X-ray irradiation (non-IR). Representative images stained with anti-pATR and anti-53BP1 antibodies, together with graphs of the percentages of positive cells, are shown in **a** and **b**, respectively. Arrowheads indicate GFP positive and pATR or 53BP1 negative cells. Data are mean ± s.e.m. (*n* = 3 and > 100 GFP+ cells). *p < 0.05, **p < 0.01, NS > 0.05. Scale bars, 50 μm

ISCs were visualized by expression of EGFP driven by the *Lgr5* promoter in Lgr5-EGFP-IRES-creERT2 mice. To investigate the DDR in ISCs, we irradiated young (2–3-month-old) and old (> 19-month-old) mice and prepared swiss roll sections of their intestines 8 h later. DNA single and double strand breaks are sensed and processed by assembling into DDR foci with DDR proteins, such as ATR, ATM, 53BP1, Chk1/2, and γH2A.X [20]. Expression of the DDR markers pATR and 53BP1 was compared between young and old ISCs with (IR) or without (non-IR) 10 gray (Gy) of X-rays irradiation in vivo by immunohistochemical analyses. The percentages of cells positive for pATR and 53BP1 were slightly increased in non-IR old ISCs and significantly lower among IR old ISCs than among IR young ISCs in the crypt base (Fig. 1). By contrast, the DDR was maintained in IR old differentiated cells in the transit amplifying compartment, whereas pATR was already induced before irradiation (Fig. 1 and Additional file 1: Figure S1). Apoptotic cleaved caspase-3-positive ISCs were observed in irradiated young crypts (Additional file 1: Figure S2). These results suggest that aging perturbs the DDR specifically in ISCs.

### DDR-associated induction of inflammatory factors is decreased in old ISCs

**Fig. 2:**
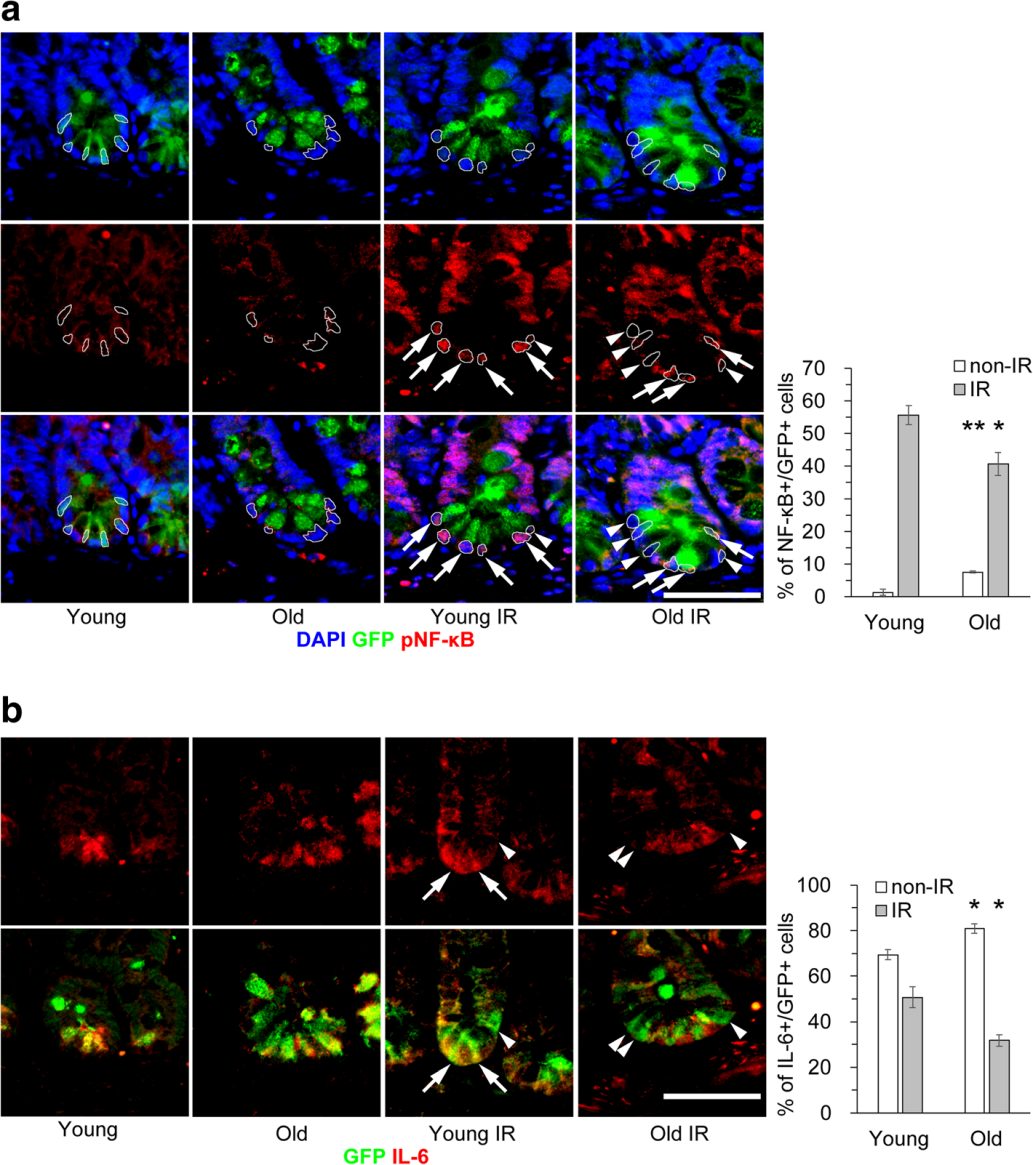
Expression of inflammatory factors is decreased in old ISCs. Immunohistochemical analyses of inflammatory factors were performed in young and old jejunum with (IR) or without 10 Gy X-ray irradiation (non-IR). Representative images stained with anti-pNF-κB and anti-IL-6 antibodies, together with graphs of the percentages of positive cells, are shown in **a** and **b**, respectively. Arrows and arrowheads indicate GFP and pNF-κB or IL-6 double-positive and GFP single-positive cells, respectively. Data are mean ± s.e.m. (*n* = 3 and > 100 GFP+ cells). *p < 0.05, **p < 0.01. Scale bars, 50 μm

We next examined the influence of aging on the induction of inflammatory factors upon DNA damage. DNA damage signaling triggers the secretion of inflammatory cytokines [21]. NF-κB is the central regulator of inflammation and inflammatory SASP factors are also induced by activation of NF-κB signaling [22]. IL-6 is a representative inflammatory cytokine and also known as a SASP factor [21]. Consistent with the findings concerning DDR markers, the percentages of cells positive for pNF-κB and IL-6 were slightly increased in non-IR old ISCs and significantly lower among IR old ISCs than among IR young ISCs (Fig. 2). Unexpectedly, IL-6 was expressed in many ISCs without irradiation and IL-6-positive ISCs were reduced by irradiation both in young and old crypts. We occasionally observed strong expression of IL-6 in lysozyme-positive Paneth cells located between two GFP-positive ISCs in old intestinal crypts (Additional file 1: Figure S3).

### DDR-associated induction of PGC-1α is decreased in old ISCs

**Fig. 3:**
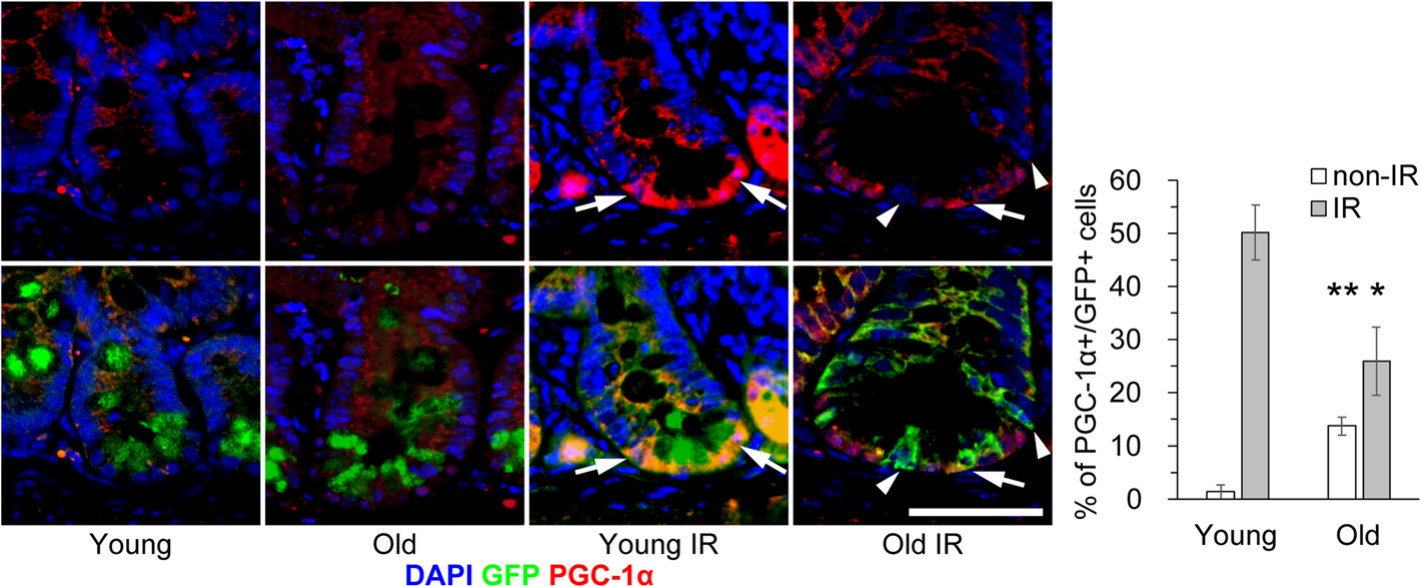
DDR-dependent mitochondrial biogenesis is decreased in old ISCs. Immunohistochemical analyses of PGC-1α were performed in young and old jejunum with (IR) or without 10 Gy X-ray irradiation (non-IR). Representative images stained with an anti-PGC-1α antibody, together with a graph of the percentage of positive cells, are shown. Arrows and arrowheads indicate GFP and PGC-1α double-positive and GFP single-positive cells, respectively. Data are mean ± s.e.m. (*n* = 3 and > 100 GFP+ cells). *p < 0.05. Scale bar, 50 μm

Mitochondrial dysfunction is associated with aging [23–25]. PGC-1α is a transcriptional coactivator and the master regulator of mitochondrial biogenesis [26, 27]. Mitochondrial biogenesis was reported to be regulated both positively and negatively upon DNA damage [25, 28–30]. PGC-1α is essential for the maintenance of homeostasis in the intestinal epithelium [31, 32]. At steady state, expression of PGC-1α is higher in villi than in crypts, and a sub-population of PGC-1α localizes to the cytoplasm [31, 33]. To investigate the influence of aging on mitochondrial biogenesis in ISCs, we investigated PGC-1α expression in irradiated mouse intestines. The percentages of cells positive for PGC-1α was slightly increased in non-IR old ISCs and significantly lower among IR old ISCs than among IR young ISCs, consistent with the findings regarding DDR-associated proteins (Fig. 3).

## Discussion

We previously demonstrated that old mesenchymal stem/stromal cells display the SASP, suggesting that this phenotype is an age-associated dysregulation of TSCs [7]. Consistent with our previous study, the percentages of cells positive for pNF-κB and IL-6 were higher among old ISCs than among young ISCs at non-IR steady state in the current study. SASP refers to the secretion of low levels of inflammatory factors by senescent cells at steady state. We investigated DDR-induced expression of inflammatory factors in ISCs. Unexpectedly, IL-6 was expressed in non-IR ISCs and IL-6-positive ISCs were reduced by irradiation both in young and old crypts. It has been reported that IL-6 has both pro- and anti-inflammatory properties and is necessary for survival and proliferation of intestinal epithelial cells by activating STAT3 signaling [34–36]. There are some reports about the induction of IL-6 after irradiation. Some of them show that the expression level of IL-6 is reduced or not altered in the short-term (several days to 3 weeks) and recovered or upregulated in long-term (4–6 weeks) after irradiation [37, 38]. On the other hand, pNF-κB was dramatically upregulated after irradiation and the percentages of pNF-κB-positive cells were lower among IR old ISCs than among IR young ISCs. These results suggest that organismal aging induces a low level of chronic inflammation at steady state, but perturbs induction of acute inflammation upon activation of the DDR.

**Fig. 4:**
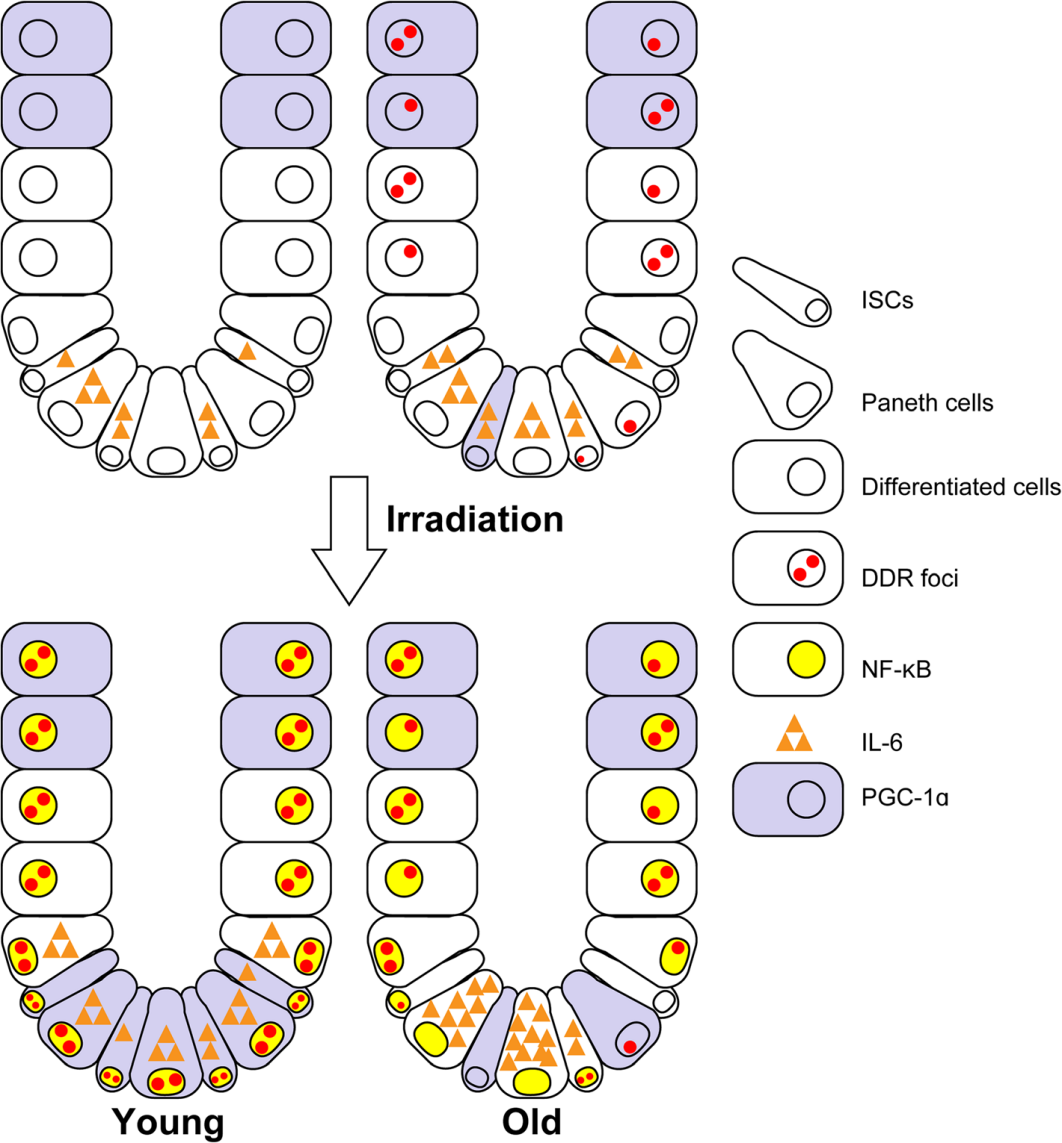
Schematic diagram of the decreases in the DDR and subsequent inflammation and mitochondrial biogenesis upon irradiation in old ISCs

As shown schematically in Fig. 4, induction of the DDR and expression of associated proteins were decreased in old ISCs. The DDR was sustained in old differentiated cells, suggesting that only the responsiveness to DNA damage was perturbed and DDR capacity was potentially sustained in old ISCs. We previously demonstrated a similar regulation for the exertion of differentiation potential in the neurogenic-to-gliogenic transition of developing neural stem/progenitor cells (NSCs) as the “competence transition” [5, 6]. NSCs are unable to respond to the gliogenic signals during early developmental stages. Both cell-intrinsic and cell-extrinsic changes can induce loss of various stem cell competence [39–41]. We also demonstrated that stem cell-intrinsic changes in competence regulation are critical for the differentiation potential and secretory profile of stem cells [6, 7, 42]. Further in vitro and in vivo investigations of both cell-intrinsic and cell-extrinsic changes in TSCs are expected to reveal the influence of the stem cell aging on organismal aging and geriatric diseases.

## Conclusions

Induction of DDR-associated proteins, inflammatory factors, and a mitochondrial biogenesis regulator following irradiation was decreased in old ISCs, but not in old differentiated cells. Our results suggest that the competence of the DDR in ISCs declines with age in vivo.

## Additional file

Additional file 1: **Figure S1.** Expression patterns of Ki-67. Proliferating progenitor cells were stained with an antibody against Ki-67 in the transit amplifying compartment. Scale bar: 50 μm. **Figure S2.** Immunohistochemical analyses of cleaved Caspase-3. GFP and cleaved Caspase-3 double-positive apoptotic intestinal stem cells (ISCs) were observed in irradiated young crypts. Arrows and arrowheads indicate GFP and cleaved Caspase-3 double-positive and GFP single-positive cells, respectively. Scale bar: 50 μm. **Figure S3.** IL-6-positive Paneth cells were observed in old crypts. Expression of IL-6 was observed in old lysozyme-positive Paneth cells in old crypts before irradiation. Arrows and an arrowhead indicate lysozyme and IL-6 double-positive and lysozyme single-positive cells, respectively. Scale bar: 50 μm. **Table S1.** List of mice. (PDF 743 kb)

## Abbreviations

DDR: DNA damage response
IBDs: Inflammatory bowel diseases
IL: Interleukin
ISCs: Intestinal stem cells
NSCs: Neural stem/progenitor cells
pATR: Phosphorylated ATR
PGC-1α: Peroxisome proliferator-activated receptor-γ coactivator 1α
pNF-κB: Phosphorylated NF-κB
SASP: Senescence-associated secretory phenotype
TSCs: Tissue-resident stem/progenitor cells
