# Supplementary material for: Age-related dysfunction of the DNA damage response in intestinal stem cells
Source: Inflamm Regen. 2019 Apr 26;39:8. doi: 10.1186/s41232-019-0096-y (PMC6485179; doi:10.1186/s41232-019-0096-y)
Supplement: Supplementary file 1 — Figure S1. Expression patterns of Ki-67. Proliferating progenitor cells were stained with an antibody against Ki-67 in the transit amplifying compartment. Scale bar: 50 μm. Figure S2. Immunohistochemical analyses of cleaved Caspase-3. GFP and cleaved Caspase-3 double-positive apoptotic intestinal stem cells (ISCs) were observed in irradiated young crypts. Arrows and arrowheads indicate GFP and cleaved Caspase-3 double-positive and GFP single-positive cells, respectively. Scale bar: 50 μm. Figure S3. IL-6-positive Paneth cells were observed in old crypts. Expression of IL-6 was observed in old lysozyme-positive Paneth cells in old crypts before irradiation. Arrows and an arrowhead indicate lysozyme and IL-6 double-positive and lysozyme single-positive cells, respectively. Scale bar: 50 μm. Table S1. List of mice. (PDF 743 kb) [file 41232_2019_96_MOESM1_ESM.pdf]

Supplementary Information

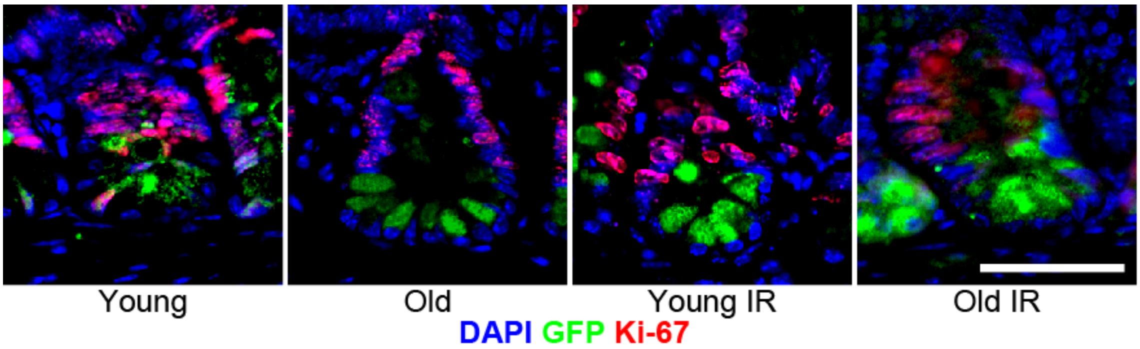

Supplementary Fig. 1 Expression patterns of Ki-67. Proliferating progenitor cells were stained with an antibody against Ki-67 in the transit amplifying compartment. Scale bar: 50 µm.

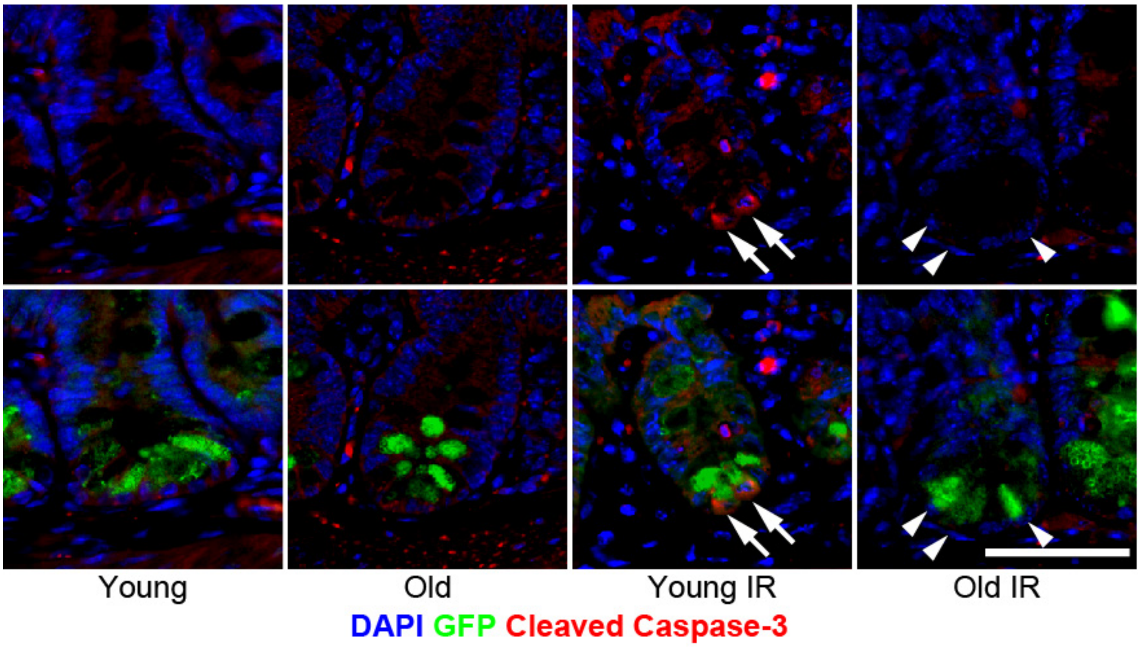

Supplementary Fig. 2 Immunohistochemical analyses of cleaved Caspase-3. GFP and cleaved Caspase-3 double-positive apoptotic intestinal stem cells (ISCs) were observed in irradiated young crypts. Arrows and arrowheads indicate GFP and cleaved Caspase-3 double-positive and GFP single-positive cells, respectively. Scale bar: 50 µm.

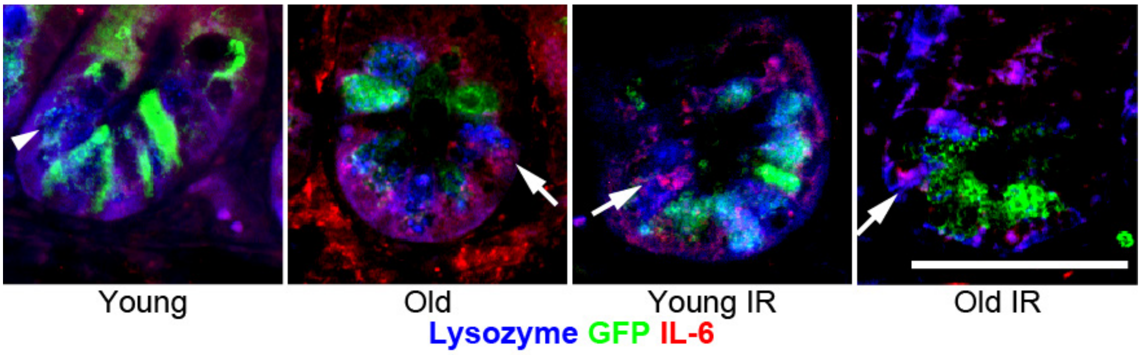

Supplementary Fig. 3 IL-6-positive Paneth cells were observed in old crypts. Expression of IL-6 was observed in old lysozyme-positive Paneth cells in old crypts before irradiation. Arrows and an arrowhead indicate lysozyme and IL-6 double-positive and lysozyme single-positive cells, respectively. Scale bar: 50 µm.

Supplementary Table 1 List of mice.

No.	Type	Sex	Birthday	X-ray	Sacrifice	Days	Weeks	Months
693	Young	♀	2017/12/11		2018/3/19	98	14	3
694	Young	♀	2017/12/11		2018/3/19	98	14	3
751	Young	♂	2018/6/8		2018/8/30	83	12	2
793	Young	♂	2018/7/10		2018/9/14	66	9	2
398	Old	♀	2016/7/22		2018/9/25	795	114	26
525	Old	♀	2017/6/24		2019/3/6	620	89	20
546	Old	♀	2017/7/23		2019/3/6	591	84	19
532	Young IR	♂	2017/7/15	2017/9/27	2017/9/27	74	11	2
534	Young IR	♂	2017/7/15	2017/9/27	2017/9/27	74	11	2
542	Young IR	♂	2017/7/23	2017/9/28	2017/9/28	67	10	2
260	Old IR	♀	2015/8/9	2017/9/28	2017/9/28	781	112	25
266	Old IR	♀	2015/9/2	2017/9/28	2017/9/28	757	108	24
182	Old IR	♀	2015/3/2	2017/9/27	2017/9/27	940	134	30
